# Nutrition and Health through the Use of Probiotic Strains in Fermentation to Produce Non-Dairy Functional Beverage Products Supporting Gut Microbiota

**DOI:** 10.3390/foods11182760

**Published:** 2022-09-08

**Authors:** Divakar Dahiya, Poonam Singh Nigam

**Affiliations:** 1 Wexham Park Hospital, Wexham Street, Slough SL2 4HL, UK; 2 Biomedical Sciences Research Institute, Ulster University, Coleraine BT52 1SA, UK

**Keywords:** beverages, nutrition, gut, health, probiotics, prebiotics, synbiotics, microbiota

## Abstract

Pure viable strains of microorganisms identified and characterised as probiotic cultures are used in the fermentation process to prepare functional beverages. The fermented probiotic products can be consumed as a source of nutrition and also for the maintenance of healthy gut microbiota. The functional beverages contain the substrates used for the preparation of product with a specific culture or a mixture of known strains used to perform the fermentation, hence these drinks can be considered as a healthy formulation of synbiotic products. If a beverage is prepared using agriculturally sourced materials, the fermented substrates with their oligosaccharides and fiber content act as prebiotics. Both the components (probiotic strain/s and prebiotic substrate) exist in a synergistic relationship in the product and contribute to several benefits for nutrition and gut health. The preparation of such probiotic beverages has been studied using non-dairy-based materials, including fruits, vegetables, nuts, grains, and cassava, a staple diet source in many regions. The consumption of beverages prepared with the use of probiotics, which contain active microbial cells and their metabolites, contributes to the functional properties of beverages. In addition, the non-dairy probiotic products can be used by consumers of all groups and food cultures, including vegans and vegetarians, and particularly consumers with allergies to dairy-based products. The aim of this article is to present a review of published research highlighting specific probiotic strains, which have the potential to enhance sustainability of healthy GIT microbiota, used in the fermentation process for the preparation of non-dairy beverages.

## 1. Introduction

The beverage industry is one of the largest manufacturing sectors in several countries; it contributes to local economies in terms of the provision of value-added products and the large workforce associated with this industry. The topic of fermented products is an essential part of the beverage industry, and it is also a traditional dietary practice in several cultures such as in South-East Asian, Far-East and African countries. In the recent decade, food and drink exports have doubled within European countries, reaching over EUR 90 billion and contributing to a positive balance of almost EUR 30 billion [1]. Scientific knowledge recently available through focused research on probiotic and synbiotic products is significantly influenced by traditional dietary customs. At the same time, the research and development sector is recognizing new opportunities for innovation and production of newer user-friendly products to cater to the wider necessities of customers for nutrition and health.

### 1.1. Health and Nutrition Potential of Functional Beverages

The intake of constituents and nutrients present in fermented products determines the growth of individual microbial strains in the microbiota of the gastrointestinal tract (GIT). Therefore, it is reasonable to consider the availability of functional and bioactive components in beverages for the regulation and determination of the gut microbiota composition [2]. Research has confirmed that the gut microbiota significantly contributes to our general well-being [3]. Alteration of the composition of the gut microbiota can affect intelligence, mood, neurodevelopment, behaviour, and psychology of its host through the gut–brain partnership [4].

There have been several studies on gut microbiota composition and its effect on health. The studies include interventions using fermented foods containing probiotics and prebiotics, with potential in their multidimensional health advantages in various systemic disorders. Beneficial gut microbiota (probiotic cultures) in the host’s GIT system selectively utilise substrates present in food as prebiotics [5]. This process supports the mechanism of increasing residence time and thus sustaining their population in the gut [6,7]. Therefore, fermented beverages must contain fermented materials (substrates used in the process) to serve as prebiotics, to ensure the growth and colonization of a large number of beneficial gut bacteria. The resident gut microbiota also act as fighters toward the exclusion of pathogenic microbiota. In addition, a healthy gut microbiome also provides other health benefits such as immune-modulatory properties and enhances the integrity of the gut barrier [8].

Several reports have produced the outcome that the gut microbiota can be targeted and manipulated by suitable dietary means [9,10]. Research findings have confirmed that the gut microbiome can be improved by the intake of functional food based on probiotics [11,12]. Probiotics and foods prepared with probiotics are generally considered safe. Probiotic cultures have been widely used in food and as additives in animal feed. These probiotic strains are easily available and accessible. The microbial strains that are widely used in the food fermentation industry are mostly LAB [13]. Their characteristics include the competitive ability to create a low pH due to acid production (lactic acid) and the production of primary and secondary metabolites. All these metabolites can play a role in the competition of LAB with other microorganisms during fermentation [14].

### 1.2. Microbial Strains Eligible for Use in Functional Food Products

The definition of probiotics according to the Food and Agriculture Organization/World Health Organization is “Live microorganisms which when administered in adequate amounts confer a health benefit on the host”, mainly through the process of replacing or including beneficial bacteria in the gastrointestinal tract [15,16]. Recently three main classes of probiotics have been proposed: 1. ‘True Probiotic’ (TP) refers to viable and active probiotic cells; 2. ‘Pseudo Probiotic’ (PP) refers to viable and inactive cells, in the forms of vegetative or spore cells (PPV or PPS); and 3. ‘Ghost Probiotic’ (GP) refers to dead/nonviable cells, in the form of intact or ruptured cells (GPI or GPR). Each class is further classified into two groups based on their site of action: internal (in vivo italics is a common way for *in vivo* and *in vitro*) or external (in vitro) [4,17,18].

Probiotics usually comprise bacteria, mainly *Lactobacillus, Bacillus*, and *Bifidobacterium*, *Streptococcus,* and *Enterococcus*, although some strains of yeast *Saccharomyces* genera have also been included in probiotic cultures. According to the International Scientific Association for Probiotics and Prebiotics (ISAPP) consensus panel, the probiotic mechanisms can be delivered by only a few strains of a particular class of bacteria, for example, *Lactobacillus casei* or *Bifidobacterium bifidum* [18]. However, to be considered efficient probiotics, the microbial strains used in food preparation or supplements must demonstrate their benefit in the host. The other properties making probiotics eligible for consumption include their role in the immune system function, and that it can be trained to identify the difference between good and pathogenic microorganisms. Probiotics help with the digestion of certain fibers, resulting in the production of health-enhancing fragments, and short-chain fatty acids [19,20].

For their beneficial properties, several GRAS strains of lactic acid bacteria have been granted a “Qualified Presumption of Safety” (QPS) status in the E.U. for food applications. Though *Lactococcus* and *Lactobacillus* have been given a GRAS status, however, the LAB genus *Streptococcus* and certain other species have been granted a GRAS/QPS status [21]. Due to their beneficial properties, LAB have been comprehensively explored in the beverage industry. The most common probiotic bacteria used for food applications are from the Lactobacillaceae family or *Bifidobacterium* genus [22,23]. This review is based on published research and reports that describe the use of GRAS probiotic microorganisms for the production of functional beverages.

## 2. Non-Dairy Probiotic Beverages for Health and Nutrition

The conventional method for making probiotic microorganisms available in the gut is through the consumption of dairy-based fermented items, normally prepared using milk as the starting raw material. The most popular fermented beverage products sold in the global market include kefir, fermented milk, and natural yogurt smoothies. Probiotic cultures are usually added to dairy products; consumers are used to the presence of microorganisms in milk-based products [2,10].

However, the consumption of such products is not suitable for groups of the population who are lactose-intolerant, allergic to milk protein, or follow a vegan diet [24,25]. Other limiting factors in the growth of dairy products with probiotics are the consumers concerned with cholesterol content, and gastroesophageal reflux disease (GERD) associated with the consumption of milk-based products. In total, 75% of the world’s population suffers from lactose intolerance [26]. According to previous studies, higher fat content in milk has shown inhibitory effects for the fermentation of *Bifidobacterium bifidum* probiotic culture in yogurt preparation. Therefore, the customers are keen on the alternative options of non-dairy-based products to meet the benefits of probiotic cultures, possibly through the intake of fermented beverages without the use of milk [27].

As a result, there has been an increasing demand for non-dairy probiotic products which meet the needs of people with dietary restrictions to dairy foods. According to a current report of the market assessed on 20 July 2022, the global dairy alternatives market is estimated to be valued at USD 27.3 billion in 2022 and is projected to reach USD 44.8 billion by 2027, recording a CAGR of 10.4% in terms of value [28]. Dairy alternatives are used in food and beverages that do not contain lactose, so such products could be suitable for the lactose-intolerant population. Globally, the health benefits of non-dairy-based products as alternatives to milk-derived preparations have taken the lead on their frequent acceptance in large-scale applications.

There are some factors guiding the economic growth of non-dairy products, such as changes in lifestyles with growing awareness for well-being, adopting of natural dietary supplements, and increasing occurrences of allergies to dairy products, and, as a result, the application areas are expanding for such probiotic–synbiotic beverages. The global market for alternatives to non-dairy products is focusing on extracts of soybean, rice, and coconut as the main matrices for the expansion of probiotic fruit and vegetable beverages [29].

### 2.1. Materials Used for the Preparation of Functional Beverages

Probiotic beverages can be made from various raw materials, such as vegetables, corn, legumes, and fruits [30,31]. Juices from a variety of fruits have been tested as an alternative nutritious material for supplementing probiotic cultures. In addition, fruit juices are a source of sugars, minerals, and vitamins for the growth of probiotic strains. The benefit of the consumption of probiotic beverages over fermented solid foods is the faster passage of liquid diets, without a longer residence time in the acidic environment of the stomach, which is a favourable factor for the higher viability of cells of probiotic strains to reach the intestine [24,31,32].

The advantage of the use of fruits and vegetables in preparation of beverages is that unlike dairy products, there are no such problems associated with the presence of lactose and cholesterol. Such non-dairy based products can be utilised by all groups of the population [32]. Fruit-based probiotic products are made from pineapple, blackberry, apple, strawberry, lemon, mango, grape, cashew, oranges, carrot, beet, etc. [26]. Pectins and fruit fibers present in the tissue of fruits such as apples, guava, bananas, and melons are potential carriers of probiotic bacteria, and act as prebiotics to provide strong adhesion support for these bacteria [33]. The use of fruits and vegetable juices in the fermentation process can increase the nutritional and functional properties of beverages, contributing beneficial effects on health [34]. The availability of probiotics in fruit and vegetable matrices provides a dietary option for non-dairy consumers [35].

The biomolecules present in fruits and vegetables improve the viability of probiotics during fermentation. The nutrients in the raw materials act as biofactors for the inclusion of microorganisms due to their functional advantages provided by their components, such as vitamins, minerals, antioxidants, and dietary fiber. These nutritional substrates make them ideal natural media for the growth of probiotics in the fermentation process. The viability of probiotics in fruit and vegetable juices depends on some important factors, such as the type of microbial strain used in fermentation, oxygen level, pH of the natural juice, temperature, and the consistence of the culture medium [36]. However, during the storage of fermented beverage products, the survival of probiotic cultures in the matrices of juices is more challenging compared to dairy-based fermented products. Despite this, studies have demonstrated that different probiotic strains are capable of growing and surviving at steady levels in fruit and vegetable beverages [37].

### 2.2. Probiotic Strains Used in Beverages Prepared from Fruits and Vegetables

The most common probiotic food products are prepared using bacteria from the Lactobacillaceae family or *Bifidobacterium* genus [38,39,40,41]. A variety of non-dairy matrices have demonstrated their potential as carriers for probiotic strains in the process of immobilization, by encapsulation or entrapment of probiotic cells on non-dairy substrates [42,43,44,45,46,47]. Table 1 summarises some studies for non-dairy beverages prepared from fruits and vegetables using selected characterised strains of probiotic cultures.

### 2.3. Probiotic Strains Used in Beverages Prepared from Grains, Seeds Beans and Tubers

The potential of probiotic yeast and lactic acid bacteria has been the focus of research in producing fermented beverages using substrates other than fruits and vegetables. The functionality and potential improvements of non-alcoholic fermented cereal beverages have been discussed in detail regarding the achievements and technological development employed to enhance the qualitative and nutritional status of fermented beverages prepared from cereals [79]. Combinations of probiotic yeast and lactic acid bacteria have been useful in the preparation of maize-based beverages [80]. In a recent study, substrates from agriculture including grains and seeds such as oat cereal, sunflower seeds, and almonds were used. The fermentation was performed using a co-culture of bacteria *Lactiplantibacillus plantarum* with the probiotic yeasts *Pichia kluyveri, Pichia guilliermondii* and *Debaryomyces hansenii* CCMA 1761 separately [81].

Studies have confirmed that the strains of probiotic microorganisms develop different flavours in media prepared using a variety of plant-based materials. The right combination of strains and substrates could have an advantage of formulating palatable probiotic products. Nine cereal-based probiotic beverages were produced by inoculating oats, barley and malt substrates with single cultures of three different strains of Lactobacillus [82].

Preparation of novel plant-based drinks have been assessed with the use of different mixtures of soy and rice milks and fermenting substrate media with single or multi-culture probiotics with several strains of Lactobacillus and Bifidobacterium [83]. The results proved that drinks prepared with the combined substrates in multi-strain fermentation generated products of higher-value. Table 2 has summarised some of such studies for non-dairy beverages prepared from beans, grains, nuts etc using selected characterized strains of probiotic cultures. The combined drinks in comparison to single substrate products had a lower amount of toxic compound furan and higher levels of desirable compounds. Multivariate analysis of volatile metabolites and physiological parameters has been suggested to assess the quality of functional plant-based drinks for industrial applications [83].

## 3. Limitations in the Use of Non-Dairy Substrates for Beverage Fermentation

In recent reports, various non dairy substrates with varying matrices have been used to deliver live probiotic microorganisms to the host. However, each substrate matrix has its unique properties and advantages, therefore, plant-based materials may imcause technological barriers [100]. Normally most probiotic strains have been iso-lated from dairy based naturally fermented products like sour milk, kefir, soft cheese etc. Therefore, there is a possibility that some of these strains may not find viabile conditions in substrates other than milk like plant based matrices and may not produce the desired optimum growth. Therefore, the application of probiotic cultures in fer-mentation to manufacture plant-based products represents a significant challenge. The suitable choice of substrate matrix and selection of the probiotic strain or the mixture of a few strains appropriate to ferment that substrate are essential factors. That strategy is necessary to ensure the success of the production of healthy food products, which are new and attractive to consumers [32,101]. 

One of the desired characteristics of probiotic cultures is their survival during their passage through the environment of gastrointestinal tract and exposure to ad-verse conditions of pH in different sections of the gut. The survival of probiotic strains in the required numbers necessary to provide probiotic impact is fundamental and it might be influenced by the non-dairy matrix components present in fermented bev-erage. In addition, the high viability of probiotic cells is also important during the stages of production and storage of probiotic products, in order to obtain the desired probiotic population and provide health benefits to the host at the time of consump-tion, which will be after a lapse of some time after their production. Therefore, some fermented beverages will have a shorter shelf-life compared to others, depending on the matrices of raw materials used in their production, whether fresh fruit and vege-tables (substrates and products summarized in Table 1), or the material used like ce-reals, seeds and nuts etc have different matrices (studies summarized in Table 2).

Though different fruit juices have been reported as a novel and appropriate growth medium for microorganisms to produce aromatic beverages from exotic and other fruits, in an effort to combine the nutritional effects with the added value bene-fits from probiotics. However, there could be a limitation based on a study where re-searchers compared the physicochemical indexes, profiles of amino acids and phenolic compounds and other volatile compounds in bog bilberry (Vaccinium uliginosum) juice fermentation conducted by a probiotic strain Lactobacillus plantarum under different pH conditions. Bilberries used in the fermentation were good fruits for the reason that they are more intensely flavoured, softer and juicier than blueberries, However, the main outcome of this study was very interesting that fermentation alters the composi-tion of substrates used in the preparation of the beverage, and that can significantly change the profile of aromatic compounds in fermented product and as a result the sensory taste qualities of beverage are compromised [102]. Thus, evaluating a balance between the probiotic starter culture and the raw materials, like fruits or vegetables of different tissue make-up, is essential to obtain a probiotic product with high sensory quality to meet the acceptance from consumers.

## 4. Conclusions and Future Perspectives

In the last few decades, there has been a rise in the number of studies on the effect of probiotic food, beverages, and fermented foods as potential synbiotics on the gut microbiome. The interest of research has moved towards clinical studies to understand how the GIT microbiome can be manipulated for establishing and maintaining a healthy gut [103]. Consumption of fermented food, beverages, or supplements as the sources of probiotics restores gut health and the regular use of probiotic products sustains the gut microbiota. The intake of foods prepared with probiotic microbial strains is recommended through the outcomes of several studies, as they have been reported to influence human health and are effective in the relief of several diseases [3,4]. Probiotic and synbiotic foods can be prepared using non-dairy substrates to meet the requirement of all groups of consumers, including vegans and the population allergic to or with low digestibility of dairy products. The global market for alternative products for lactose intolerant consumers in the form of non-dairy products was valued at USD 8.51 billion in 2016. With the popularity of such products, the consumption market is estimated to increase to approximately USD 24.6 billion by 2025 [104], and is projected to reach USD 44.8 billion by 2027, recording a CAGR of 10.4% in terms of value [28]. Therefore, the development of new technologies that are more economical and the use of substrates of suitable matrices for probiotics viability and survival are extremely important for the supply of non-dairy probiotic foods to meet demand. Although there is great potential for the use of fruit extracts [105] as probiotic beverages, there is the potential for commercial-scale probiotic drinks production from cheaply available seasonal fruits.

Innovation for new products depends on the fact that the matrices of non-dairy substrates sourced from plants have different characteristics that could limit the viability of probiotic strains when compared to milk-based matrices. The selection of suitable fruits or vegetables for beverage production needs to be considered, as some extracts may contain anti-nutritional factors, unfavorable pH, and lower nutrient availability. Future work could investigate and compare the performance of lactic acid bacteria with fermentation by probiotic yeasts and bacteria in co-cultivation. This could offer more options for the use of other non-dairy cheaper agricultural substrates such as seeds, grains, nuts, and cereals for the commercial-scale production of non-dairy fermented beverages for vegan consumers. Further studies on fermentation profile, the viability of probiotic strains during their refrigerated storage, the production of desirable volatile or undesirable non-volatile compounds, and the resultant sensory profile of the beverage will support the use of a variety of agriculturally sourced substrates other than fruits and vegetables.

## Figures and Tables

**Table 1:** List of microorganisms used for the preparation of fruit- and vegetable-based non-dairy probiotics beverages.

No.	Microbial Strains	Non-Dairy Products	Reference
1.	*L. plantarum, L. casei, Bifidobacterium animalis* subsp. *lactis*	Probiotic non-fermented blended beverages with banana, strawberry, and juçara or palmiteiro fruit (*Euterpe edulis* Martius)	[46]
2.	Microencapsulated *Bifidobacteria*	Passionfruit juice in a functional non-dairy product for probiotic delivery	[47]
3.	*L. salivarius* spp. *salivarius* encapsulated	Probiotic culture incorporated into a fruit matrix	[48]
4.	*Lacticaseibacillus rhamnosus, Lacticaseibacillus paracasei subsp. paracasei, Lactobacillus acidophilus, Bifidobacterium animalis* subsp. *lactis*, *Lactiplantibacillus plantarum*	Functional fermented juice of a mixture of pineapple, spinach, cucumber, pumpkin, and Jerusalem artichoke juices	[49]
5.	*Lactobacillus casei* (commercial lyophilised culture)	Fruit juice from sweet oranges (*Citrus sinensis*)	[50]
6.	*Lactobacillus plantarum*	Cashew apple juice, a functional beverage with sweet aroma and reduced astringency	[51]
7.	*Lactobacillus acidophilus*	Beet and orange mixed juices (1:1 and 1:2 *v*/*v*) with 28 days shelf life	[52]
8.	*Lacticaseibacillus casei* with prebiotics inulin, oligofructose, and polydextrose	Water-soluble extract of baru almond	[53]
9.	*Lactobacillus fermentum*—ATCC 9338	Prickly pears (*Opuntia* sp.) juice	[54]
10.	*Lactobacillus casei* NRRL B-442	Cantaloupe melon and cashew fruit (*Anacardium occidentale* L.) juice	[55]
11.	*Lactobacillus plantarum* DW12	Mature coconut water functional fermented beverage	[56]
12.	*Lactobacillus rhamnosus* GR-1	Probiotic mixed fruit beverage (apple cider, orange, grapes) juices fortified with short- or long-chain inulin fiber	[57]
13.	*Lactobacillus paracasei* K5 isolated from Greek Feta-type cheese	Functional symbiotic pomegranate beverage	[58]
14.	*Lactobacillus brevis, L. plantarum, L. rhamnosus, Fructobacillus tropaeoli*	Cherimoya (*Annona cherimola* Mill.) fermented juice matrix for the formulation of stable functional beverages	[59]
15.	*Lactobacillus rhamnosus* GG	Mixed pineapple (*Ananas comosus* L. Merril) and Jussara (Euterpe edulis Martius) beverage	[60]
16.	*Lactobacillus rhamnosus* ATCC 7469	Juices extracted from guava fruit	[61]
17.	*Bifidobacterium lactis* Bb12, *Lactobacillus plantarum* 299V, *Lactobacillus acidophilus* La5	Probiotic beverage made from pineapple juice	[62]
18.	*Lactobacillus plantarum* ATCC 8014	Fermented watermelon juice with or without supplementation with inulin or fructo-oligosaccharide	[63]
19.	*Lactobacillus acidophilus, L. plantarum*, and *L. delbrueckii*	Pomegranate juice alone and blended with kokum-rind extract (*Garcinia indica choisy*)	[64]
20.	*L. plantarum, L. brevis, L. paracasei, L. fermentum, L. pentosus*	Fruit juice from apple, orange, and grapes	[65]
21.	*Lactobacillus helveticus* L10, *Lactobacillus paracasei* L26, *Lactobacillus rhamnosus* HN001)	Carambola (starfruit, Averrhoa carambola) juice	[66]
22.	*L. plantarum*	Guichang (Kiwi fruit (*Actinidia Lindl*. spp.))	[67]
23.	*Lactobacillus casei* ATCC 393, *Lactobacillus plantarum*	Malolactic fermentation of fruit juices	[68]
24.	*Lactobacillus plantarum,* *Lactobacillus fermentum, Lactobacillus paracasei*	Fruit processing by-products potential for application as novel probiotics	[69]
25.	Probiotic yeasts *Pichia kudriavzevii*, *Wickerhamomyces subpelliculosus*	Cornelian cherry (*Cornus mas* L.) functional beverage	[70]
26.	*Lactiplantibacillus plantarum*	Passionfruit juice	[71]
27.	*Lactobacilli*	Cherry juice fermentation	[72]
28.	*S. cerevisiae*	Pomegranate (*Punica granatum* L.)	[73]
29.	Lactobacillus casei	Pomegranate juice	[74]
30.	*Lactiplantibacillus plantarum*	Red jujube fruits and bamboo shoots fermented	[75]
31.	Two strains of *Lactobacillus plantarum*	Vegetable and fruit beverage of apples, pears, and carrots	[76]
32.	*Lactobacillus casei* L4,	Fermented coconut water beverage	[77]
33.	*Lactobacillus plantarum* PMO 08	Extract from tomato pulp	[78]

**Table 2:** List of microorganisms used for the preparation of beverages from beans, grains, nuts and tubers.

No.	Microbial Strains	Non-Dairy Products	Reference
1.	*Bifidobacterium lactis, Lactobacillus helveticus*, *L. actobacillus paracasei*, yeast *Lindnera saturnus* (*Williopsis saturnus* var. *saturnus*)	Cocultured functional probiotic beverage okara (soya bean residue) with enhanced nutritional and aroma profiles	[84]
2.	*Lactobacillus rhamnosus* GG	Peanut and soya bean water-soluble extracts	[85]
3.	co-culture LAB + probiotic Yeasts *Lactiplantibacillus plantarum, Pichia kluyveri, P. guilliermondii, Debaryomyces hansenii*	Blend of almonds, oats, and sunflower seeds, a vegan probiotic drink	[81]
4.	*L. rhamnosus* GG, a single culture of *Saccharomyces boulardii* CNCM-I745	Fermented coffee brews with bioactive components and antioxidant capacities retained	[86]
5.	*Lactobacillus plantarum* CCMA 0743 (from Cauim) and *Torulaspora delbrueckii* CCMA 0235 (from Tarubá), and the commercial probiotic, *L. acidophilus* LAC-04	Cassava (*Manihot esculenta* Crantz) and rice-based beverage with functional properties	[87]
6.	*Lactobacillus fermentum* CCMA 0215 with yeast strains (*Torulaspora delbrueckii* CCMA 0234,0235, *Pichia caribbica* CCMA 0198, *Saccharomyces cerevisiae* CCMA 0232, 0233)	Functional cassava fermented beverage	[88]
7.	commercial probiotic *Lactobacillus acidophilus* LACA 4, *Lactobacillus plantarum* CCMA 0743, *Torulaspora delbrueckii* CCMA 0235	Maize-blended rice beverages	[89]
8.	*Lacticaseibacillus casei* with prebiotics inulin, oligofructose, and polydextrose	Water-soluble extract of baru almonds	[53]
9.	Binary culture of *Pediococcus acidilactici* and *L. acidophilus*; co-culture of *P. acidilactici*, *L. acidophilus, S. cerevisiae*	Peanut/soya milk functional beverage	[90]
10.	*Lactiplantibacillus plantarum, Lacticaseibacillus casei* Shirota (as control)	*Amaranthus hypochondriacus* L. a pseudocereal	[91]
11.	*Lactobacillus plantarum* L7	Rice-based fermented beverage “Bhaati Jaanr”	[92]
12.	*Lactobacillus fermentum* KKL1	Rice-based fermented beverage	[93]
13.	*Lactobacillus plantarum* DSM 9843	Quinoa beverage	[94]
14.	*Lactiplantibacillus plantarum* CIDCA 8327, *Lacticaseibacillus paracasei* BGP1	Soya-based fermented beverage	[95]
15.	*Lactobacillus* sp., *Streptococcus thermophilus, Bifidobacterium animalis* subsp. *lactis* and *Propionibacterium*	Rice-based yogurt-type milk substitutes	[96]
16.	*Limosilactobacillus fermentum* MG7011	Rice-based probiotic beverages	[97]
17.	Lactic acid bacteria from kimchi.	Rice-based yogurt with various beans	[98]
18.	*Lactobacillus acidophilus* La-5, *Bifidobacterium animalis* Bb-12 in co-culture with *Streptococcus thermophilus*	Synbiotic fermented beverage from soya milk of vegetable soyabeans	[99]
19.	*Lactobacillus paracasei* LBC-81, probiotic yeasts, *Saccharomyces cerevisiae* CCMA 0731, *S. cerevisiae* CCMA 0732, *Pichia kluyveri* CCMA 0615	Functional corn-based beverage	[80]
20.	*Lactobacillus fermentum*, *L. plantarum, L. helveticus, Bifidobacterium bifidum*, and *B. longum*	Soya and rice drinks	[83]
21.	*Lactobacillus acidophilus* (NCIMB 8821), *Lactobacillus plantarum* (NCIMB 8826), *Lactobacillus reuteri* (NCIMB 11951)	Oats, barley and malt beverages	[82]

## Data Availability

Not applicable.
